# Sustained Efficacy and Safety of Tildrakizumab in Psoriasis Vulgaris Despite Multiple Prolonged Treatment Interruptions: A Case Report

**DOI:** 10.7759/cureus.87927

**Published:** 2025-07-14

**Authors:** Kazuki Yatsuzuka, Jun Muto, Satoshi Yoshida, Ken Shiraishi, Yasuhiro Fujisawa

**Affiliations:** 1 Department of Dermatology, Ehime University Graduate School of Medicine, Toon, JPN

**Keywords:** biologics, interleukin-23, psoriasis vulgaris, tildrakizumab, treatment interruption

## Abstract

Psoriasis vulgaris is a persistent inflammatory skin disease that often necessitates long-term biologic therapy to maintain symptom control and prevent flares. Interruptions in biologic treatment are sometimes unavoidable due to individual health concerns or external circumstances, but the impact of repeated, extended gaps in therapy remains insufficiently understood. We describe a case involving a woman with moderate-to-severe psoriasis vulgaris who achieved sustained remission using tildrakizumab, even after multiple treatment interruptions, each lasting over a year. The patient initially received various conventional and biologic treatments before transitioning to tildrakizumab. Despite prolonged discontinuations prompted by personal concerns, she consistently regained complete skin clearance within months of restarting therapy. No therapy-related adverse events were recorded during any biologic treatment period. This case emphasizes the potential resilience of tildrakizumab efficacy in the setting of intermittent administration, suggesting its suitability for patients who may face unavoidable gaps in care. Further research is warranted to evaluate immunogenicity risks and long-term effectiveness in larger, diverse patient populations.

## Introduction

Psoriasis is a chronic, immune-mediated skin disease characterized by erythematous, scaly plaques that can appear on various parts of the body, most commonly the scalp, elbows, knees, and lower back. Among its subtypes, psoriasis vulgaris (plaque-type psoriasis) is the most prevalent form. This condition not only affects the skin but can also have significant psychosocial and systemic impacts, including an association with psoriatic arthritis, cardiovascular disease, and metabolic syndrome. Treatment of moderate-to-severe psoriasis vulgaris typically aims to reduce inflammation, control symptoms, and improve patients’ quality of life. Therapeutic strategies include topical agents, phototherapy, conventional systemic therapies such as methotrexate or cyclosporine, and, more recently, biologic therapies that target specific pathways involved in the disease’s pathogenesis. Biologics, particularly those that inhibit interleukin (IL)-23 and IL-17, have transformed the management of psoriasis vulgaris by providing sustained disease control with a favorable safety profile.

Patients with moderate-to-severe psoriasis vulgaris often require lifelong biologic therapy; however, extended treatment interruptions may become necessary due to life events such as surgery, infections, pregnancy, or childbirth. Although some clinical trials have shown that efficacy can be regained after a single treatment interruption [1], the effects of multiple, long-term interruptions remain unclear. Such interruptions may increase the risk of forming anti-drug antibodies (ADAs). To our knowledge, this may be the first real-world case of psoriasis vulgaris in which tildrakizumab (IL-23 inhibitor) maintained efficacy and safety despite multiple treatment interruptions lasting over one year.

## Case presentation

**Figure 1:**
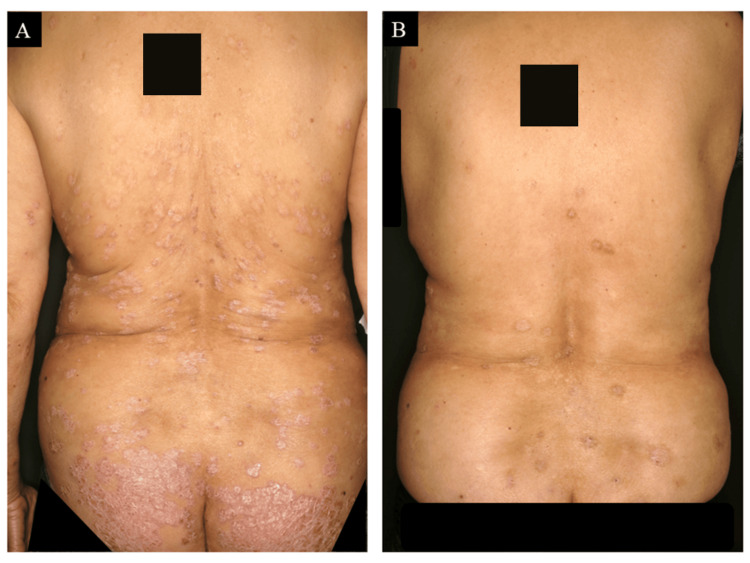
Cutaneous manifestations before and after ustekinumab treatment. Before ustekinumab initiation, numerous scaly erythematous plaques were widely distributed across the extremities and trunk (A). Significant improvement of skin lesions after ustekinumab initiation (B).

A 52-year-old female patient, a smoker with hypertension and dyslipidemia, developed psoriasis vulgaris. Conventional therapies, including topical corticosteroids, vitamin D₃ analogs, narrowband ultraviolet B therapy, and oral etretinate, failed to provide adequate control. At the age of 77 years, ustekinumab was initiated (baseline Psoriasis Area and Severity Index (PASI) score: 21) (Figure 1A), with the PASI score improving to 0.4 within four months. She maintained a PASI score between 0 and 1.5 on ustekinumab every 12 weeks (Figure 1B).

**Figure 2:**
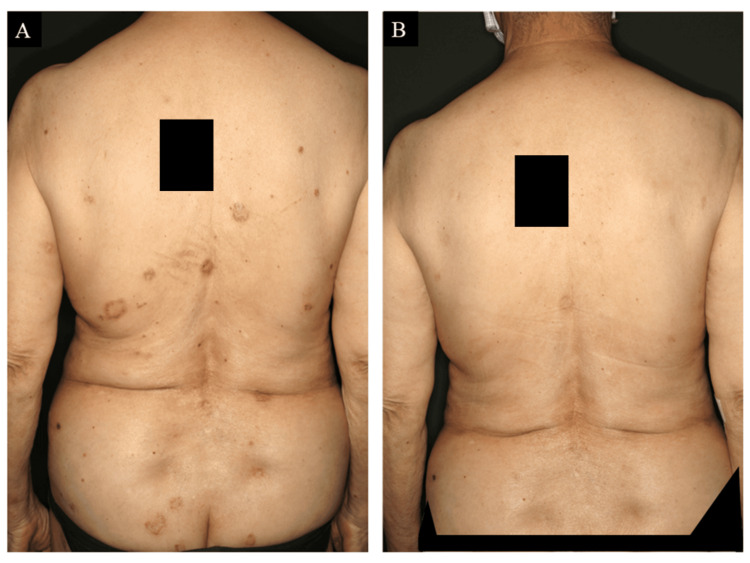
Cutaneous manifestations before and after tildrakizumab treatment. One year after discontinuing ustekinumab, her skin lesions flared (A). Significant improvement in skin lesions was achieved within seven months of initiating tildrakizumab (B).

However, two years and eight months after initiating ustekinumab, she voluntarily discontinued follow-up due to concerns about an increased risk of infection during the COVID-19 pandemic. Ten months after discontinuation, her skin lesions flared, and one year later, her PASI score had worsened to 12.6 (Figure 2A). She subsequently returned to our department. She requested a switch to a newer agent, and tildrakizumab was initiated, resulting in complete PASI clearance within seven months (Figure 2B).

**Figure 3:**
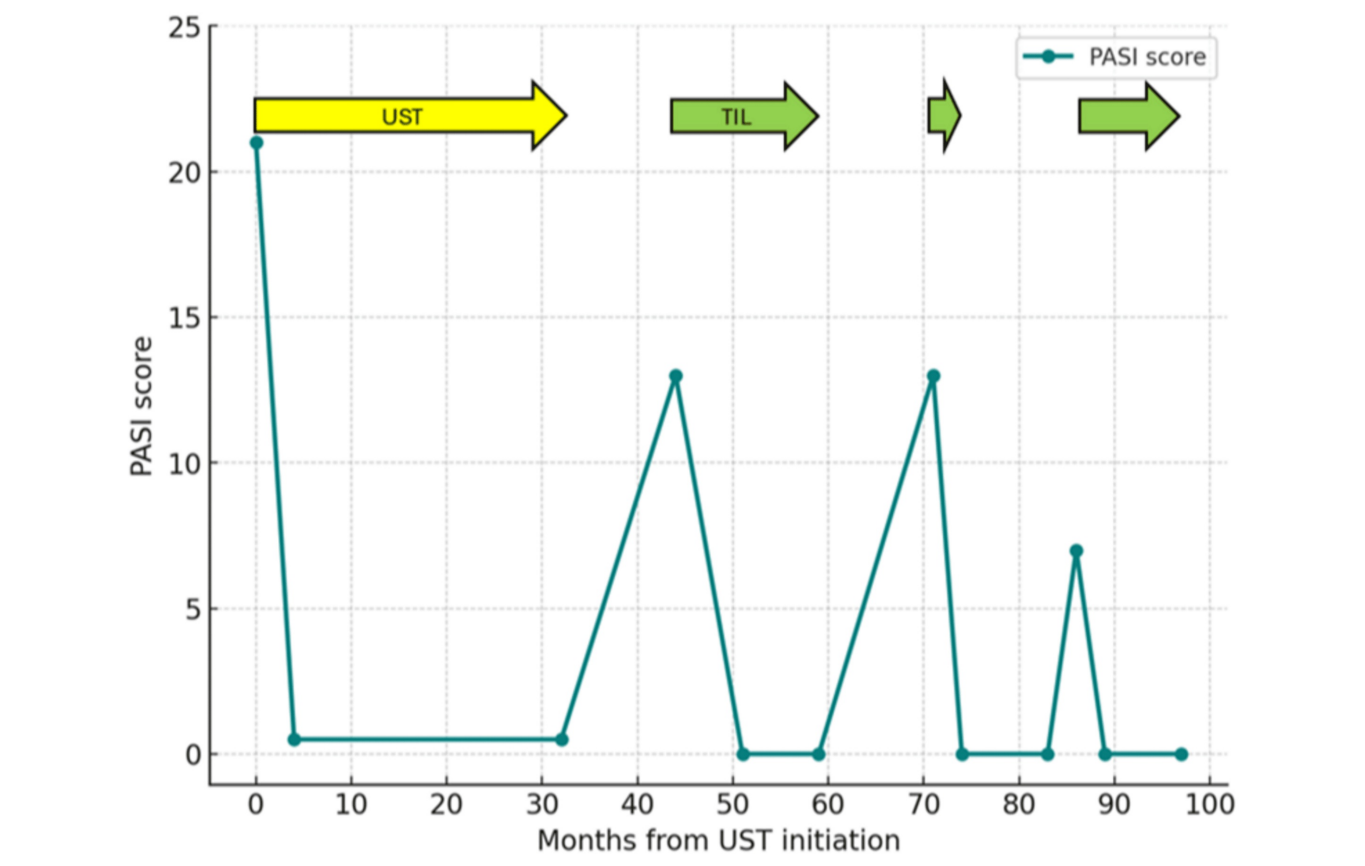
Overview of treatment timeline and changes in the PASI score. PASI: Psoriasis Area and Severity Index; UST: ustekinumab; TIL: tildrakizumab

Upon patient request, the dosing interval was extended to every 16 weeks, with continued clearance. However, 15 months after initiation, she chose to stop treatment due to concerns about potential side effects and again withdrew from clinical visits. One year later, with a PASI score of 12.6, tildrakizumab was resumed, and complete clearance was achieved within three months. After another discontinuation, again due to concerns about side effects, she returned a year later with a PASI score of 7. Tildrakizumab was restarted without a loading dose, and complete clearance was achieved after a single injection at week 12. She was later diagnosed with Alzheimer’s disease and now receives tildrakizumab every 12 weeks under nursing facility supervision, maintaining complete PASI clearance for eight months without adverse events (Figure 3).

## Discussion

The development of ADAs is a known concern in patients receiving biologic therapies for psoriasis. Monoclonal antibody aggregates may serve as danger signals to antigen-presenting cells, thereby enhancing antigen processing and triggering T-cell-mediated immune responses [2]. This process can lead to the formation of ADAs [2]. Although some studies found no clear correlation between ADA formation and reduced clinical efficacy, other reports indicate that high ADA levels may be associated with diminished therapeutic response [3]. Moreover, ADA formation might be linked to serious adverse events such as thromboembolism and vasculitis-like reactions [4].

Continuous biologic therapy is generally considered more effective and safer in preventing both disease flares and ADA formation [5,6]. Intermittent dosing has been associated with a higher risk of ADA formation compared to continuous administration [6]. Nevertheless, real-world situations, such as infections, comorbidities, or patient preferences, often necessitate treatment pauses. Our patient experienced two separate tildrakizumab treatment interruptions, each lasting over one year. Notably, no adverse events were observed, and the efficacy remained unchanged over time. Fully human or humanized monoclonal antibodies, such as tildrakizumab, tend to have lower immunogenicity, which may result in a lower likelihood of ADA formation [2,4], even with intermittent dosing.

Although a few psoriasis cases involving repeated biologic reinitiations have been reported [7], to our knowledge, no real-world data have demonstrated both sustained efficacy and safety after more than two one-year treatment gaps, as in our case. While the ADAs could not be assessed due to the unavailability of commercially validated assays, our case underscores tildrakizumab’s potential as a flexible and durable option for psoriasis patients with complex care needs. Further prospective studies are warranted to evaluate immunogenicity profiles, long-term efficacy, and safety outcomes associated with the use of intermittent biologics in broader populations.

## Conclusions

This case illustrates the sustained efficacy and safety of tildrakizumab in a patient with psoriasis vulgaris despite multiple prolonged treatment interruptions, each lasting over one year. Remarkably, disease control was rapidly regained following each reinitiation, with no apparent loss of response or adverse events, even without a loading dose. These findings suggest that tildrakizumab, a humanized monoclonal antibody targeting IL-23, may exhibit low immunogenicity, enabling durable efficacy despite intermittent dosing. To our knowledge, this is the first reported real-world case demonstrating successful long-term management of psoriasis vulgaris with repeated tildrakizumab reinitiations after extended gaps in therapy. This case highlights the potential flexibility of tildrakizumab for patients who require temporary discontinuation due to comorbidities, concerns about adverse events, or personal circumstances. Although this case offers valuable insights, it is important to acknowledge that the inherent limitations of case reports include a potential risk of reporting bias. Further investigation in larger, prospective cohorts is necessary to confirm these observations, assess immunogenicity, and define optimal strategies for managing treatment interruptions in psoriasis care.
